# Application of Sodium Silicate Enhances Cucumber Resistance to Fusarium Wilt and Alters Soil Microbial Communities

**DOI:** 10.3389/fpls.2018.00624

**Published:** 2018-05-11

**Authors:** Xingang Zhou, Yanhui Shen, Xuepeng Fu, Fengzhi Wu

**Affiliations:** ^1^Department of Horticulture, Northeast Agricultural University, Harbin, China; ^2^Key Laboratory of Biology and Genetic Improvement of Horticultural Crops (Northeast Region), Ministry of Agriculture, Harbin, China

**Keywords:** cucumber, sodium silicate, microbial communities, induced resistance, Fusarium wilt

## Abstract

Exogenous silicates can enhance plant resistance to pathogens and change soil microbial communities. However, the relationship between changes in soil microbial communities and enhanced plant resistance remains unclear. Here, effects of exogenous sodium silicate on cucumber (*Cucumis sativus* L.) seedling resistance to Fusarium wilt caused by the soil-borne pathogen *Fusarium oxysporum* f.sp. *cucumerinum* Owen (FOC) were investigated by drenching soil with 2 mM sodium silicate. Soil bacterial and fungal community abundances and compositions were estimated by real-time PCR and high-throughput amplicon sequencing; then, feedback effects of changes in soil biota on cucumber seedling resistance to FOC were assessed. Moreover, effects of sodium silicate on the growth of FOC and *Streptomyces* DHV3-2, an antagonistic bacterium to FOC, were investigated both *in vitro* and in the soil environment. Results showed that exogenous sodium silicate enhanced cucumber seedling growth and resistance to FOC. In bare soil, sodium silicate increased bacterial and fungal community abundances and diversities. In cucumber-cultivated soil, sodium silicate increased bacterial community abundances, but decreased fungal community abundances and diversities. Sodium silicate also changed soil bacterial and fungal communality compositions, and especially, decreased the relative abundances of microbial taxa containing plant pathogens but increased these with plant-beneficial potentials. Moreover, sodium silicate increased the abundance of *Streptomyces* DHV3-2 in soil. Soil biota from cucumber-cultivated soil treated with sodium silicate decreased cucumber seedling Fusarium wilt disease index, and enhanced cucumber seedling growth and defense-related enzyme activities in roots. Sodium silicate at pH 9.85 inhibited FOC abundance *in vitro*, but did not affect FOC abundance in soil. Overall, our results suggested that, in cucumber-cultivated soil, sodium silicate increased cucumber seedling resistance to Fusarium wilt by changing soil microbial communities rather than by directly inhibiting the growth of FOC.

## Introduction

Silicon (Si) is the second most abundant mineral element in soil and comprises about 28% of the Earth’s crust (Ye et al., 2013). Although functions of Si in plant physiology and biochemical aspects have long been debated, the beneficial effects of this element for plant growth, development, and resistance to both abiotic and biotic stresses have been well-documented in many plant species (Ma, 2004; Reynolds et al., 2016; Liu et al., 2017; Wang et al., 2017). For example, exogenous silicates (e.g., potassium silicate, calcium silicate, and sodium silicate) can increase plant resistance to diseases such as powdery mildew and Fusarium wilt in cucumber (*Cucumis sativus* L.) (Liang et al., 2005; Safari et al., 2012), sheath blight and blast in rice (*Oryza sativa* L.) (Ye et al., 2013; Zhang et al., 2013) and powdery mildew in wheat (*Triticum aestivum* L.) (Guével et al., 2007).

Several mechanisms have been proposed to explain the enhanced resistance in plants by exogenous silicates. First, the polymerization of Si beneath the cuticle and in the cell walls increases the physical barrier to pathogens (Ye et al., 2013; Liang et al., 2015; Debona et al., 2017). Second, Si plays a metabolic role in the host–pathogen interaction by enhancing the activities of plant defensive enzymes, leading to increased accumulation of defensive compounds such as phenolics and phytoalexins to improve plant resistance to biotic and abiotic stresses (Ma, 2004; Reynolds et al., 2016). Third, Si can induce systemic resistance in plants (Debona et al., 2017). For example, application of silicates (potassium silicate or colloidal silicon dioxide) to roots induced systemic resistance in rice (*Oryza sativa* L.) and tomato (*Solanum lycopersicum* L.) (Ye et al., 2013; Kurabachew and Wydra, 2014).

The soil microbial community is one of the main components determining soil health and is considered a major driver of plant defense to belowground pathogens (Mendes et al., 2013). Plant-beneficial microorganisms (e.g., some species in *Pseudomonas* and *Streptomyces* spp.) can protect plants directly by inhibiting plant pathogens and indirectly by inducing systemic resistance in plants (Zhao et al., 2012; Pieterse et al., 2014). Induced plant defenses are regulated by highly interconnected signaling networks, in which plant hormones such as jasmonic acid, salicylic acid, and ethylene are key regulators (Pieterse et al., 2014). Activation of the jasmonic acid signaling pathway altered rhizosphere microbial communities and caused shifts in abundances of bacteria with plant pathogens-suppression potentials (Carvalhais et al., 2013). Wang et al. (2013) demonstrated that potassium silicate changed soil microbial community structure and activity. However, whether changes in soil microbial communities induced by exogenous silicate play some role in enhancing plant resistance to pathogens remains unclear.

Mineral nutrients can directly affect the physiological status of plant pathogens and their antagonists (Qin and Tian, 2005). For example, zinc and copper inhibited the plant pathogenic fungus *Fusarium oxysporum* f.sp. *ciceri* to produce fusaric acid, a phytotoxin; but promoted *Pseudomonas fluorescens* 4-92, an antagonist to this pathogen, to produce 2, 4-diacetylphloroglucinol, a secondary metabolite with antifungal activity (Saikia et al., 2009). *In vitro* tests also demonstrated that sodium silicate inhibited the mycelial growth of some fungi, such as *Alternaria alternata*, *Fusarium semitectum*, and *Trichothecium roseum* (Bi et al., 2006). Therefore, there is a possibility that exogenous silicates can protect plants through inhibiting plant pathogens and/or stimulating plant-beneficial microorganisms in the soil.

Fusarium wilt of cucumber, caused by the soil-borne pathogen *F. oxysporum* f.sp. *cucumerinum* (FOC), is a serious vascular disease worldwide (Zhou and Wu, 2009). Previous studies revealed that sodium silicate enhanced cucumber resistance to Fusarium wilt, and potassium silicate changed soil microbial communities (Safari et al., 2012; Wang et al., 2013). We hypothesized that (1) changes in the soil biota caused by sodium silicate enhanced cucumber seedling resistance to Fusarium wilt, and (2) sodium silicate could inhibit FOC while stimulate its antagonist. The objectives of this study were (1) to elucidate the responses of soil microbial communities to exogenous sodium silicate, (2) to assess whether changes in soil microbial communities induced by sodium silicate exert feedback effects on the resistance of cucumber seedlings to Fusarium wilt, and (3) to evaluate the effects of exogenous sodium silicate on FOC and *Streptomyces* DHV3-2, a bacterium antagonistic to FOC, both *in vitro* and in soil.

## Materials and Methods

### Pot Experiment

Soils used in this study were collected from the upper soil layer (0–15 cm) of an open field in the experimental station of Northeast Agricultural University, Harbin, China (45°41′ N, 126°37′ E). The soil was a black soil (Mollisol) with sandy loam texture: soil organic matter, 74 g kg^-1^; available N, 101 mg kg^-1^; Olsen P, 48 mg kg^-1^; available K, 124 mg kg^-1^; available Si, 32 mg kg^-1^; pH (1:2.5, w/v), 5.85; EC (1:2.5, w/v), 0.41 mS cm^-1^. Analytically pure sodium silicate (Na_2_SiO_3_⋅9H_2_O), purchased from Basifu, Co. Ltd., Tianjin, China, was used in this study.

Cucumber seeds (cv. ‘Jinyan 4,’ susceptible to FOC) were soaked in water at 55°C for 30 min and then germinated in sand in a growth chamber at 26°C. After emergence, cucumber seedlings with two cotyledons were transplanted into pots (16 cm in diameter, 14 cm in height) containing 1 kg of dry weight soils and maintained in a greenhouse (28°C day/18°C night, relative humidity of 60–80%, 16 h light/8 h dark). Each pot contained a single plant. Then, pots containing cucumber seedlings and pots without cucumber seedlings (bare soil) were treated with nutrient solution with or without 2.0 mM sodium silicate. The applied sodium silicate concentration into the soil was based on the results of Liang et al. (2005). In total, there were four treatments in this experiment: cucumber-cultivated soil treated with sodium silicate (CSi) or without sodium silicate (C), bare soil treated with sodium silicate (BSi) or without sodium silicate (B). The nutrient solution used contained macronutrients (in mM): 1.0 Ca(NO_3_)_2_, 0.9 KNO_3_, 0.3 MgSO_4_, and 0.1 KH_2_PO_4_; EDTA-buffered micronutrient solution (in μM): 2.5 MnSO_4_, 1.0 CuSO_4_, 10 ZnSO_4_, 1.0 CoSO_4_, 1.0 NiCl_2_, and 115.5 EDTANa_2_; and other micronutrient (in μM): 35 NaCl, 10 H_3_BO_3_, 0.05 Na_2_MoO_4_. Sodium silicate was added to achieve a concentration of 2.0 mM sodium silicate. The pH of the nutrient solution buffered with 0.1 mM HEPES was adjusted to 7.5 with 0.05 mM NaOH. For treatments applied with sodium silicate (CSi and BSi), 100 ml of the above nutrient solution with 2.0 mM sodium silicate was added per pot weekly. For treatments without sodium silicate (C and B), 100 ml of the above nutrient solution without 2.0 mM sodium silicate was added per pot weekly.

The experiment was set up following a randomized block design with three replicate blocks. In each block, there were 30 pots per treatment. During the experiment, the pot was weighed to ensure that the water content was maintained at about 60% of its water holding capacity, and the position of these pots in each plot was randomly changed every 2 days.

### Plant Dry Biomass Measurement and Soil Sampling

At 10, 20, and 30 days after the first application of sodium silicate, three cucumber seedlings in each triplicate of each treatment were harvested. The plant dry weight was measured after oven drying at 70°C to constant weight.

Soil samples were collected at 30 days after the first application of sodium silicate as described previously (Zhou and Wu, 2012). Samples from nine pots per replicate of each treatment were mixed to obtain a composite sample. A portion of the fresh sampled soils was used for plant–soil feedback experiments; the other portion was stored at -80°C for DNA extraction.

### DNA Extraction and Real-Time PCR Analysis of Bacterial and Fungal Community Abundances

Total soil DNA was extracted from 0.28 g soil samples using the PowerSoil DNA Isolation Kit (MO BIO Laboratories, Carlsbad, CA, United States) according to the manufacture’s protocol.

Soil bacterial and fungal community abundances were estimated by measuring bacterial 16S rRNA gene and fungal internal transcribed spacer (ITS) region numbers with primer sets of 338F/518R (Muyzer et al., 1993) and ITS1F/ITS4 (White et al., 1990; Gardes and Bruns, 1993), respectively. SYBR Green-based real-time PCR (qPCR) analysis was performed with an IQ5 real-time PCR system (Bio-Rad Lab, Hercules, CA, United States) as described before (Zhou et al., 2017). Standard curves were created with a 10-fold dilution series of plasmids containing the 16S rRNA gene or ITS region. The threshold cycle (Ct) value obtained for each sample was compared with the standard curve to determine the initial copy number of the target gene.

### Illumina Miseq Sequencing of Bacterial and Fungal Communities

The compositions of soil bacterial and fungal communities were analyzed with Illumina MiSeq sequencing. Primer sets of 515F/907R and ITS1F/ITS2 were used for bacterial and fungal communities, respectively, as described before (Ren et al., 2015; Zhou et al., 2017). Each sample was amplified in triplicate, and the amplicons were purified, quantified, and pyrosequenced using a MiSeq Genome Sequencer PE300 Titanium platform (Majorbio Bio-Pharm Technology, Co. Ltd., Shanghai, China).

Raw FASTQ files were de-multiplexed, quality-filtered and processed using QIIME (version 1.17) (Caporaso et al., 2010), and the paired reads were joined with FLASH (Magoč and Salzberg, 2011). The joined pairs were then quality filtered with multiple steps, such as removal of sequences <220 bp with ambiguous base ‘N’ and average base quality score <20. Operational taxonomic units (OTUs) with 97% similarity cut off were clustered using UPARES (version 7.1) (Edgar, 2013) and annotated through BLAST in SILVA (bacteria) and Unite (fungi) databases, and chimeric sequences were identified and removed using USEARCH in QIIME (Caporaso et al., 2010). After filtering reads by basal quality control and removing singleton OTUs, 645,943 quality bacterial sequences and 761,849 quality fungal sequences were obtained in total. The average read lengths were 397 and 252 bp for the 16S rRNA gene and ITS region, respectively. The data set was deposited in the NCBI-Sequence Read Archive with the submission Accession No. SRP118555.

### Inoculation of FOC and Fusarium Wilt Disease Index Measurement

A strain of FOC was isolated and identified from a Fusarium-wilted cucumber plant grown in a greenhouse. FOC was grown on potato-dextrose-agar (PDA) medium and conidia were obtained as described before (Zhou and Wu, 2009). At the two-leaf stage, cucumber seedlings, which were prepared as describe in the pot experiment, were inoculated with a 2 × 10^8^ conidia ml^-1^ suspension of FOC according to the method of Fortunato et al. (2014). First, cucumber seedlings were carefully removed from soil, and roots were cut off with a sterilized scissor and then dipped in the FOC conidial suspension for 20 min. Seedlings dipped in sterilized water were used as control. Then, cucumber seedlings were transferred back to their original pots. Finally, 20 ml of the FOC conidial suspension was drenched to the soil surface containing FOC-treated seedlings per pot. Each treatment contained 15 seedlings and was done in triplicates.

All cucumber seedlings were harvested on 15 days after the inoculation of FOC. Roots were washed with deionized water to remove soil residues and then treated with a solution of 2% ascorbic acid for 10 min to avoid tissue oxidation. The disease severity was recorded using a scale containing six grades as suggested by Liu et al. (1995).

### Plant–Soil Feedback Experiment

The feedback effects of soil biota on cucumber seedling growth and resistance to FOC were evaluated through the addition of soil inoculum method as described before (Brinkman et al., 2010; Zhou et al., 2017, 2018). Sterilized soils collected from the open field were used as background soils, while soils treated with or without sodium silicate from the pot experiment were used as inocula. The ratio of inoculum-to-background soil was 6% (mass/mass). Briefly, soils collected from the open field were sterilized with three cycles of autoclaving (121°C, 30 min) and cooling to room temperature. Then, these background soils were mixed with different inocula. There were four treatments: sterilized open field soils mixed with (1) bare soil treated without sodium silicate, (2) bare soil treated with sodium silicate, (3) cucumber-cultivated soil treated without sodium silicate, and (4) cucumber-cultivated soil treated with sodium silicate. To maintain a relatively sterile working environment, all pots and tools were sterilized.

Cucumber seedlings with two cotyledons were transplanted into pots (16 cm in diameter, 14 cm in height) with 1 kg of prepared soils. There were three replicates for each treatment, and 33 pots per replicates, including 27 pots for inoculation of FOC and six pots for measuring cucumber seedling dry biomass. Then, seedlings were maintained in a greenhouse (28°C day/18°C night, relative humidity of 60–80%, 16 h light/8 h dark). All pots were randomly arranged. These seedlings were watered with sterile water three times a week and the soil moisture was maintained at about 50% of its water holding capacity.

#### Inoculation of FOC and Measurements of Defense-Related Enzyme Activities and Fusarium Wilt Disease Index

Seven days after transplanting, cucumber seedlings from the plant–soil feedback experiment were inoculated with FOC as described above. Then, cucumber seedling root samples from three plants per replicate were collected on 0, 3, 6, and 9 days after FOC inoculation. Root samples were stored in liquid nitrogen during sampling and transferred to -80°C until further analysis.

Superoxide dismutase (SOD, EC1.15.1.1) activity was measured by the nitroblue tetrazolium (NBT) method at 560 nm by calculating the photoreduction of NBT (Beauchamp and Fridovich, 1971). The reaction was initiated by placing tubes below two 15-W fluorescent lamps for 10 min. Peroxidase (POD, EC1.11.1.7) activity was assayed as described by Bernt et al. (1974), and one unit of enzyme activity was defined as the amount of enzyme necessary to increase the absorbance by one unit per minute. Phenylalanine ammonia-lyase (PAL, EC4.3.1.24) activity was determined spectrophotometrically by determining the production of *trans*-cinnamic acid from L-phenylalanine at 290 nm (Mozzetti et al., 1995). PAL activity was expressed as nanomoles per minute per mg of protein. β-1,3-glucanase (GLU, EC3.2.1.6) activity was determined as described by Lever (1972). The absorbance of the product released by GLU was measured at 540 nm, and the activity of GLU was expressed in absorbance units per minute per mg of protein.

On 15 days after the inoculation of FOC, the Fusarium wilt disease severity was measured by recording 15 seedlings per replicate of each treatment as described above.

#### Measurement of Plant Dry Biomass

At 10 and 20 days after transplanting, three cucumber seedling plants per replicate from the plant–soil feedback experiment were harvested. Cucumber seedling plant dry weights were measured after oven drying to constant weight at 70°C.

### Effects of Sodium Silicate on the Growths of FOC and *Streptomyces* DHV3-2

#### *In Vitro* Experiment

FOC conidia was obtained on PDA medium and *Streptomyces* DHV3-2 spores, an antagonist of FOC, on Gause’s synthetic agar medium. Then, FOC and *Streptomyces* DHV3-2 were inoculated into potato dextrose and Gause’s synthetic liquid media in 250 ml flasks, respectively, and incubated for 7 days at 28°C with shaking at 120 rpm.

As the growth of microorganisms were sensitive to pH (Bi et al., 2006), sodium silicate were applied under two pH conditions: (1) the pH of sodium silicate solution adjusted to 6.32 with 0.1 M HCl and (2) the pH of sodium silicate solution not adjusted (pH 9.85). Potato dextrose and Gause’s synthetic liquid media containing 0.2 mM sodium silicate were prepared. Meanwhile, sterilized water (pH 6.32) was used a control. Then, effects of sodium silicate on FOC and *Streptomyces* DHV3-2 were tested under the following conditions: (1) FOC in potato dextrose liquid medium, (2) *Streptomyces* DHV3-2 in Gause’s synthetic liquid medium, (3) both FOC and *Streptomyces* DHV3-2 in potato dextrose liquid medium, and (4) both FOC and *Streptomyces* DHV3-2 in Gause’s synthetic liquid medium. The final concentrations of both FOC and *Streptomyces* DHV3-2 were 10^4^ CFU ml^-1^. The numbers of FOC and *Streptomyces* DHV3-2 were counted using a hemocytometer in each medium 3 day after inoculation. There were three replicates for each treatment, and five flasks per replicates.

#### Microcosm Experiment in Soil

Soils taken from the open field were dried at room temperature for 4–5 days and then sieved (2 mm) and autoclaved as described above. Ten milliliter of 10^3^ CFU ml^-1^ of FOC or *Streptomyces* DHV3-2 were inoculated into 100 g of dry weight soils, which were filled in 200 ml jars. The soil water content was maintained at 60% of its water holding capacity. These jars were incubated at 28°C in the dark for 14 days to allow FOC and *Streptomyces* DHV3-2 abundances reach at stable levels (data not shown). Then, these jars were treated with 10 ml of 2.0 mM sodium silicate solution. Meanwhile, sterilized water (pH 6.32) was used a control. Seven and fourteen days after sodium silicate application, FOC and *Streptomyces* DHV3-2 abundances in the soil were measured using the plate counting method with Komada medium and Gao 1 medium, respectively. There were three replicates for each treatment, and five jars per replicates.

### Statistical Analysis

To assess microbial diversity among samples in a comparable manner, a normalized dataset was used for subsequent analysis. In the normalized data, the lowest number of sequences in all samples (43,748 for bacteria and 52,108 for fungi) were randomly selected. The alpha diversity indices (ACE, Chao1, Shannon, and Simpson indices) were calculated with the ‘vegan’ package in ‘R’ (R Core Team, 2017). For beta diversity, principal coordinates analysis (PCoA) was performed to determine differences in microbial community structures based on Bray–Curtis distances. Analysis of similarities (ANOSIM) with the Bray–Curtis distance and 999 permutations was carried out to test for differences in microbial community compositions. Heat map was used to show the relative abundances of dominant classified bacterial (average relative abundance > 0.5%) and fungal (average relative abundance > 0.3%) genera. Relative abundances of microbial taxa were tested for differences among treatments using the Kruskal–Wallis non-parametric test and Dunn test for *post hoc* comparisons at the 0.05 probability level with the ‘PMCMR’ package in ‘R’ (R Core Team, 2017).

Data were analyzed by analysis of variance (ANOVA). For data of Fusarium wilt disease index from the pot experiment, FOC and *Streptomyces* DHV3-2 abundances from the microcosm experiment, mean comparison between treatments was performed based on the Student’s *t*-test at the 0.05 probability level; for other data, mean comparison between treatments was performed based on the Tukey’s honestly significant difference (HSD) test at the 0.05 probability level with SAS software (version 8.0, SAS Institute, Cary, NC, United States).

## Results

### Cucumber Seedling Growth and Fusarium Wilt Disease Index

**FIGURE 1:**
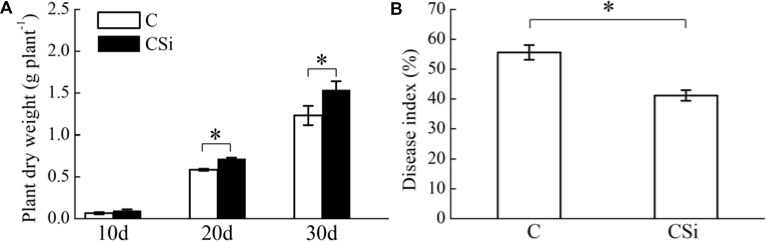
Effects of sodium silicate on cucumber seedling growth **(A)** and Fusarium wilt disease index **(B)**. CSi and C represent cucumber-cultivated soil treated with and without sodium silicate, respectively. Asterisks indicate significant difference between treatments at the 0.05 probability level (Student’s *t*-test).

Without inoculation of FOC, cucumber seedling dry weight was significantly increased by exogenous sodium silicate after 20 and 30 days (*P* < 0.05) (**Figure 1A**). After 21 days of FOC inoculation, cucumber seedling Fusarium wilt disease index was significantly decreased by exogenous sodium silicate (*P* < 0.05) (**Figure 1B**).

### Soil Bacterial and Fungal Community Abundances

**FIGURE 2:**
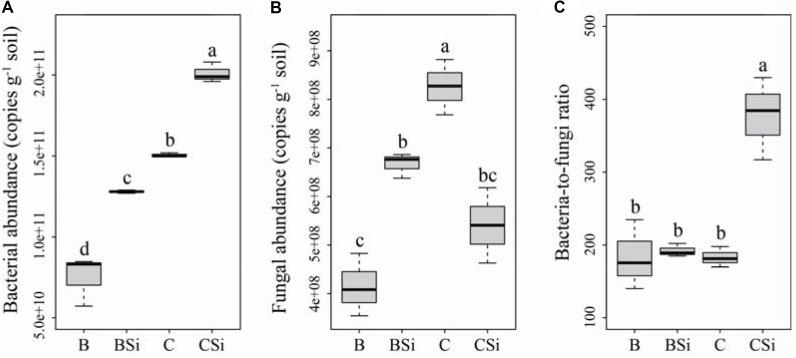
Soil bacterial **(A)** and fungal **(B)** community abundances, and bacterial-to-fungi ratio **(C)** as determined by real-time PCR. BSi and B represent bare soil treated with and without sodium silicate, respectively. CSi and C represent cucumber-cultivated soil treated with and without sodium silicate, respectively. Values with different letters are significantly different at the 0.05 probability level (Turkey’s HSD test).

In bare soil, sodium silicate significantly increased soil bacterial and fungal community abundances (**Figures 2A,B**, *P* < 0.05), but had no effect on the bacteria-to-fungi ratio (**Figure 2C**). In cucumber-cultivated soil, sodium silicate significantly increased soil bacterial community abundance but decreased soil fungal community abundance, and thus increased the bacteria-to-fungi ratio (**Figure 2**) (*P* < 0.05).

### Soil Bacterial Community Composition and Structure

**FIGURE 3:**
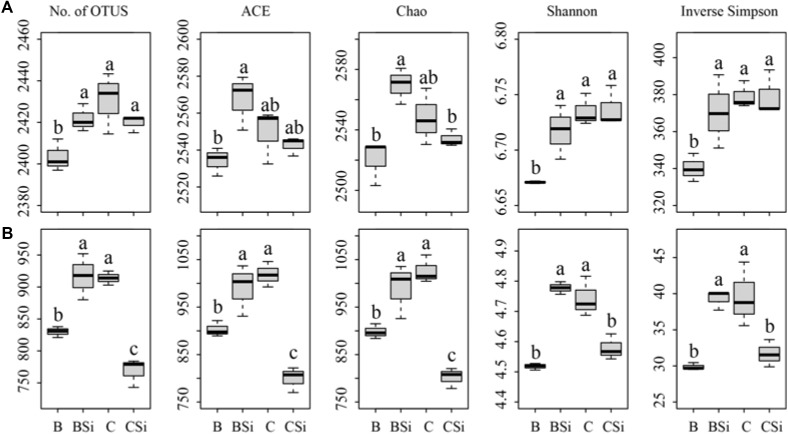
Soil bacterial **(A)** and fungal **(B)** community diversity indices as determined by Illumina Miseq sequencing. OTUs were delineated at 97% similarity. The number of OTUs (No. of OTUs), ACE, Chao, Shannon, and Inverse Simpson indices of the bacterial and fungal communities were calculated from 43,748 bacterial and 52,108 fungal sequences per sample, respectively. BSi and B represent bare soil treated with and without sodium silicate, respectively. CSi and C represent cucumber-cultivated soil treated with and without sodium silicate, respectively. Values with different letters are significantly different at the 0.05 probability level (Turkey’s HSD test).

**FIGURE 4:**
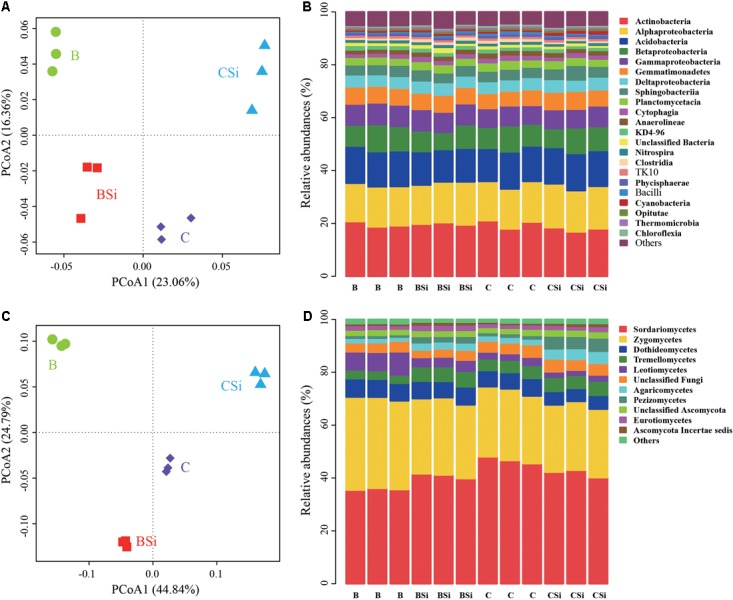
Principal coordinates analysis (PCoA) analysis of soil bacterial **(A)** and fungal **(C)** communities and relative abundances of main soil bacterial **(B)** and fungal **(D)** classes. OTUs were delineated at 97% similarity. The PCoA plots were based on the Bray–Curtis distances at the OTU level. For both bacterial and fungal communities, classes with average relative abundances >0.5% were shown. BSi and B represent bare soil treated with and without sodium silicate, respectively. CSi and C represent cucumber-cultivated soil treated with and without sodium silicate, respectively.

In bare soil, the treatment of sodium silicate had higher number of observed OTUs, ACE, Chao, Shannon, and Inverse Simpson indices of the bacterial community than the treatment without sodium silicate (*P* < 0.05) (**Figure 3A**). However, in cucumber-cultivated soil, sodium silicate had no significant effects on these bacterial community diversity indices. PCoA analysis at the OTU level separated the four treatments from each other (**Figure 4A**), and the difference was statistically significant (ANOSIM, *R* = 0.873, *P* = 0.001).

At the phylum level, *Proteobacteria*, *Actinobacteria*, and *Acidobacteria* were the dominant phyla (average relative abundances > 10%) in all samples (Supplementary Figure S1A). *Actinobacteria*, *Alphaproteobacteria*, and *Acidobacteria* were the top three classes in all sample, and they accounted for about 47.93% of the total bacterial sequence (**Figure 4B**). In bare soil, sodium silicate increased the relative abundances of bacterial phylum *Firmicutes*, bacterial classes *Clostridia*, *Bacilli*, and *Thermomicrobia* (*P* < 0.05). In cucumber-cultivated soil, sodium silicate increased the relative abundances of bacterial phylum *Gemmatimonadetes* and bacterial class *Cyanobacteria*, but decreased the relative abundances of phylum *Firmicutes*, classes *Anaerolineae*, *Clostridia*, *Bacilli*, and *Thermomicrobia* (*P* < 0.05).

At the genus level, more than 530 bacterial taxa were detected across all samples (data not shown). In bare soil, sodium silicate increased the relative abundances of *Pseudarthrobacter*, *Microlunatus*, and *Streptomyces* spp., but decreased the relative abundance of *Nocardioides* spp. (*P* < 0.05) (Supplementary Figure S2A). In cucumber-cultivated soil, sodium silicate increased the relative abundances of *Gemmatimonas*, *Rhizomicrobium*, and *Rhodanobacter* spp., but decreased the relative abundances of *Pseudarthrobacter* and *Microlunatus* spp. (*P* < 0.05).

### Soil Fungal Community Composition and Structure

In bare soil, the treatment of sodium silicate had higher number of observed OTUs, ACE, Chao, Shannon, and Inverse Simpson indices of the fungal community than the treatment without sodium silicate (*P* < 0.05) (**Figure 3B**). However, in cucumber-cultivated soil, sodium silicate had opposite effects on these indices. PCoA analysis applied to visualize the differences among samples showed a clear separation among treatments (**Figure 4C**). ANOSIM also demonstrated that the fungal communities were significantly different among treatments (*R* = 0.982, *P* = 0.001).

At the phylum level, all samples were dominated by *Ascomycota*, *Zygomycota* and *Basidiomycota*, and they accounted for about 95.06% of the total fungal sequence (Supplementary Figure S1B). In bare soil, sodium silicate increased the relative abundances of *Ascomycota* and *Basidiomycota*, and decreased the relative abundance of *Zygomycota* (*P* < 0.05). In cucumber-cultivated soil, sodium silicate increased the relative abundance of *Basidiomycota*, and decreased the relative abundance of *Ascomycota* (*P* < 0.05).

At the class level, *Sordariomycetes* and *Zygomycetes* were the top two classes in all samples, and they accounted 70.05% of the fungal sequences (**Figure 4D**). In bare soil, sodium silicate increased the relative abundances of *Sordariomycetes*, *Tremellomycetes*, *Agaricomycetes*, *Pezizomycetes*, *Eurotiomycetes* and *Ascomycota Incertae sedis*, and decreased the relative abundances of *Zygomycetes* and *Leotiomycetes* (*P* < 0.05). In cucumber-cultivated soil, sodium silicate increased the relative abundances of *Agaricomycetes*, *Pezizomycetes* and *Ascomycota Incertae sedis*, and decreased the relative abundances of *Sordariomycetes* and *Dothideomycetes* (*P* < 0.05).

At the genus level, more than 410 fungal taxa were detected (data not shown). In bare soil, sodium silicate increased the relative abundances of *Chaetomium*, *Gibellulopsis*, *Cryptococcus*, *Guehomyces*, *Kernia*, *Acremonium*, *Aspergillus*, *Cladosporium*, and *Pseudeurotium* spp., but decreased the relative abundances of *Mortierella*, *Humicola*, *Stagonosporopsis*, *Nectria*, *Phaeomycocentrospora*, *Preussia*, and *Exophiala* spp. (*P* < 0.05) (Supplementary Figure S2B). In cucumber-cultivated soil, sodium silicate increased the relative abundances of *Gibberella*, *Fusarium*, *Guehomyces*, *Aspergillus*, *Athelopsis* and *Pseudeurotium* spp., but decreased the relative abundances of *Pseudallescheria*, *Ilyonectria*, *Acremonium*, *Myrothecium*, *Stagonosporopsis*, *Metarhizium*, *Phaeomycocentrospora*, *Preussia*, *Cephaliophora*, *Cladosporium*, and *Cercophora* spp. (*P* < 0.05).

### Effects of Soil Biota on Cucumber Seedling Growth and Resistance to Fusarium Wilt

**FIGURE 5:**
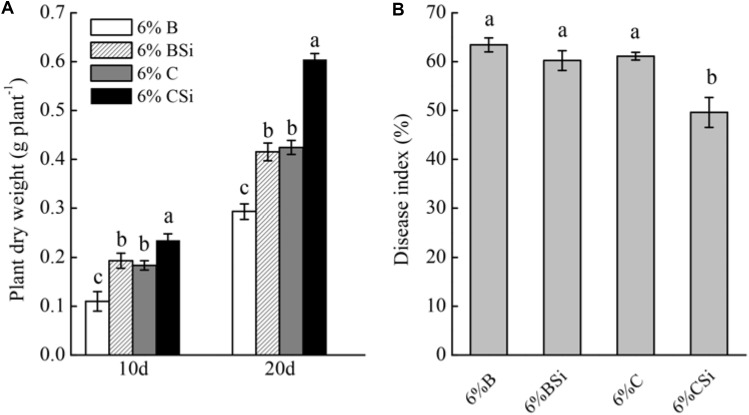
Feedback effects of changes in soil biota induced by sodium silicate on cucumber seedling growth **(A)** and Fusarium wilt disease index **(B)**. 6%B and 6%BSi represent sterilized open field soil mixed with 6% (mass/mass) bare soil treated without and with sodium silicate, respectively. 6%C and 6%CSi represent sterilized open field soil mixed with 6% (mass/mass) cucumber-cultivated soil treated without and with sodium silicate, respectively. Values with different letters are significantly different at the 0.05 probability level (Turkey’s HSD test).

Dry weight of seedlings grown in background soil mixed with bare soil treated with sodium silicate was significantly higher than that of seedlings grown in background soil mixed with bare soil treated without sodium silicate (*P* < 0.05) (**Figure 5A**). Meanwhile, dry weight of seedlings grown in background soil mixed with cucumber-cultivated soil treated with sodium silicate was significantly higher than that of seedlings grown in background soil mixed with cucumber-cultivated soil treated without sodium silicate (*P* < 0.05).

Cucumber seedling Fusarium wilt disease index was similar between treatments of background soil mixed with bare soil treated with and without sodium silicate (**Figure 5B**). However, background soil mixed with cucumber-cultivated soil treated with sodium silicate had significantly lower disease index than that mixed with cucumber-cultivated soil treated without sodium silicate (*P* < 0.05).

**FIGURE 6:**
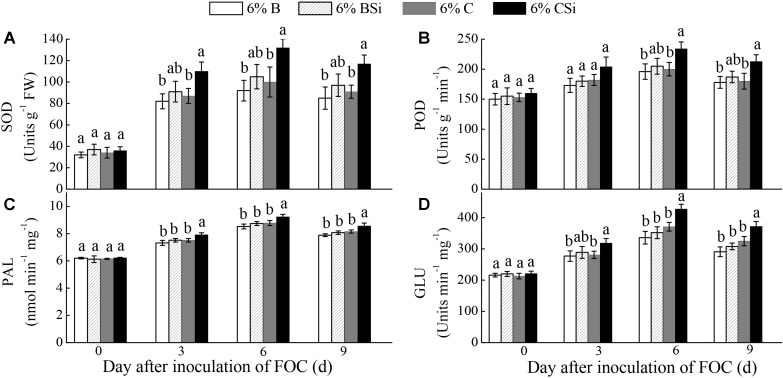
Feedback effects of changes in soil biota induced by sodium silicate on cucumber seedling root SOD **(A)**, POD **(B)**, PAL **(C)**, and GLU **(D)** activities. 6%B and 6%BSi represent sterilized open field soil mixed with 6% (mass/mass) bare soil treated without and with sodium silicate, respectively. 6%C and 6%CSi represent sterilized open field soil mixed with 6% (mass/mass) cucumber-cultivated soil treated without and with sodium silicate, respectively. Values with different letters are significantly different at the 0.05 probability level (Turkey’s HSD test).

Before inoculation of FOC, all treatments had similar activities of SOD, POD, PAL, and GLU in cucumber seedling roots(**Figure 6**).Activities of SOD, PAL, and GLU on 3, 6, 9 days after inoculation of FOC(**Figures 6A,C,D**), and activity of POD on 6 and 9 days after inoculation of FOC (**Figure 6B**) were higher in background soil mixed with cucumber-cultivated soil treated with sodium silicate than that treated without sodium silicate (*P* < 0.05). However, background soils mixed with bare soil treated with and without sodium silicate had similar activities of these enzymes (**Figure 6**).

### Effects of Sodium Silicate on Growths of FOC and *Streptomyces* DHV3-2

**FIGURE 7:**
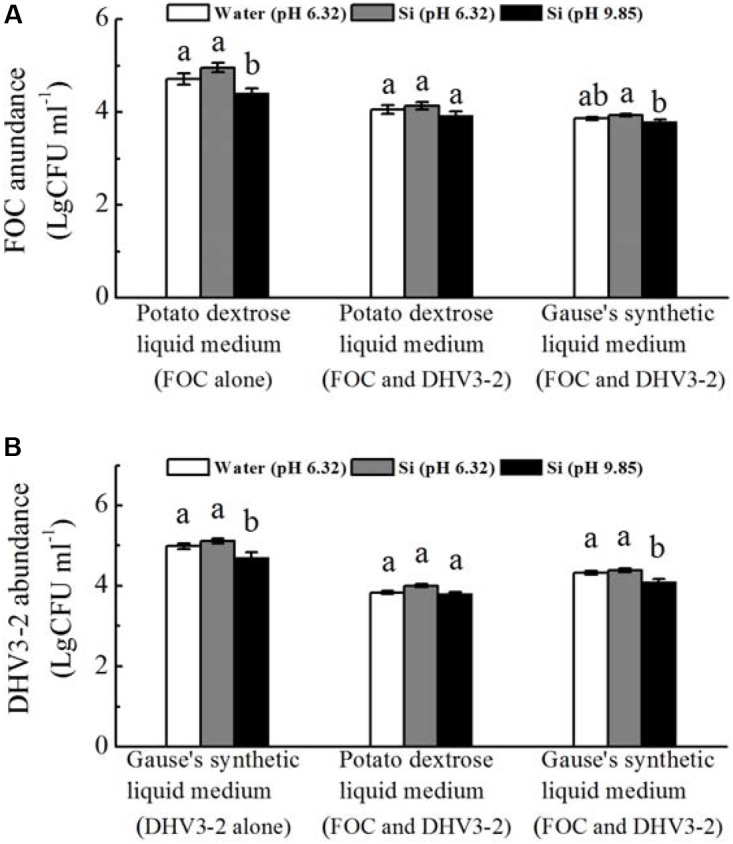
Effects of sodium silicate on FOC **(A)** and *Streptomyces* DHV3-2 **(B)** *in vitro*. FOC and *Streptomyces* DHV3-2 were grown alone or together in Potato dextrose and Gause’s synthetic liquid medium. Si (pH 6.32) and Si (pH 9.85) represent the treatments of sodium silicate solution with pH adjusted (6.32) or not (9.85), respectively. Water (pH 6.32) represents the treatment of water with a pH of 6.32. CFU, colony forming unit. Values with different letters are significantly different at the 0.05 probability level (Turkey’s HSD test).

*In vitro* experiments revealed that, when grown alone, spore numbers of FOC and *Streptomyces* DHV3-2 were significantly decreased by sodium silicate (pH not adjusted) (*P* < 0.05) (**Figure 7**). However, sodium silicate with pH adjusted have no effect on spore numbers of FOC or *Streptomyces* DHV3-2.

When FOC and *Streptomyces* DHV3-2 were grown together in the potato dextrose liquid medium, spore numbers of FOC or *Streptomyces* DHV3-2 were not affected by sodium silicate with and without pH adjusted (**Figure 7**). When FOC and *Streptomyces* DHV3-2 were grown together in Gause’s synthetic liquid medium, spore numbers of FOC and *Streptomyces* DHV3-2 were significantly decreased by sodium silicate without pH adjusted (*P* < 0.05). However, the treatment of sodium silicate with pH adjusted did not affect FOC and *Streptomyces* DHV3-2 in Gause’s synthetic liquid medium when these two microorganisms grown together.

**FIGURE 8:**
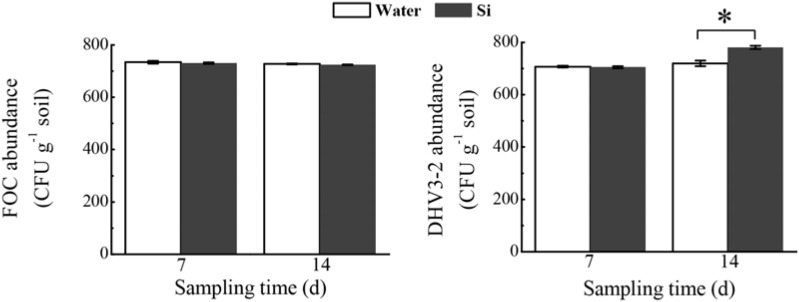
Effects of sodium silicate on FOC and *Streptomyces* DHV3-2 in the soil environment. Water and Si represent the treatments of water and sodium silicate, respectively. CFU, colony forming unit. Asterisks indicate significant difference between treatments at the 0.05 probability level (Student’s *t*-test).

In the microcosm experiment, sodium silicate had no effect on the FOC abundance, but significantly increased *Streptomyces* DHV3-2 abundance (*P* < 0.05) (**Figure 8**).

## Discussion

It was demonstrated that root and foliar applications of silicates can promote plant growth and offer protection against plant pathogens (Guével et al., 2007; Safari et al., 2012; Liang et al., 2015; Debona et al., 2017). Similarly, this study found that sodium silicate increased cucumber plant dry weight and decreased cucumber Fusarium wilt disease index. Soil microorganisms play pivotal roles in modulating plant growth and health through mutualistic and pathogenic effects, and regulation of nutrient cycling (Berendsen et al., 2012; Pieterse et al., 2014). Both *in vivo* and *in vitro* studies showed that exogenous silicates could affect soil microorganisms (Bi et al., 2006; Wang et al., 2013). In this study, high-throughput amplicon sequencing and real-time PCR revealed that sodium silicate changed soil bacterial and fungal community diversities, compositions, and abundances.

Recent studies found that changes in microbial communities could influence plant performance (Brinkman et al., 2010; Zhou et al., 2017, 2018). Our plant–soil feedback experiments showed that changes in soil biota in both bare soil and cucumber-cultivated soil induced by sodium silicate had positive feedback effects on cucumber seedling growth. Moreover, changes in soil biota in cucumber-cultivated soil induced by sodium silicate decreased cucumber seedling Fusarium wilt disease index and increased activities of defense enzymes in cucumber roots (except for POD activity on 3 days after inoculation of FOC). However, changes in soil biota in bare soil induced by sodium silicate did not affect cucumber seedling Fusarium wilt disease index and root defense enzyme activities. These suggested that changes in soil biota induced by sodium silicate enhanced cucumber seedling resistance to Fusarium wilt in cucumber-cultivated soil but not in bare soil. Therefore, our first hypothesis was partially supported. However, it should be noted that most previous studies showed that silicates reduced disease incidence through enhancing plant resistance to pathogen infection (Ma, 2004; Liang et al., 2015; Reynolds et al., 2016). The sodium silicate-induced changes in soil microbial communities may be one possible mechanism and other mechanisms could not be excluded.

Except for distinct feedback effects of changes in soil biota induced by sodium silicate between in bare soil and cucumber-cultivated soil as discussed above, sodium silicate also had differing effects on soil microbial communities in bare soil and cucumber-cultivated soil. For example, sodium silicate increased soil bacterial community diversity indices in bare soil but had no effects on these indices in cucumber-cultivated soil. Moreover, sodium silicate increased the abundance and diversity indices of soil fungal community in bare soil while decreased these parameters in cucumber-cultivated soil. Root exudates exhibit a multitude of functions in ecological interactions with microbial soil communities, including acting as carbon resources, signaling molecules, attractants and stimulants, and inhibitors or repellents (Baetz and Martinoia, 2014; Ge et al., 2017). Kidd et al. (2001) found that root application of silicic acid changed the composition of root exudates from maize (*Zea mays* L.), which contributed to the enhanced aluminum resistance in silicic acid-treated maize. Therefore, it is possible that the observed differences in changes in soil microbial communities and their feedback effects on cucumber Fusarium wilt resistance between the treatments of bare soil and cucumber-cultivated soil may attribute to presence of cucumber in the cucumber-cultivated soil. Future studies should focused on elucidating the effects of sodium silicate on cucumber root exudates and its relationship with changes in soil microbial communities.

Previous studies demonstrated that exogenous silicates (such as sodium silicate, sodium metasilicate, and potassium silicate) can protected plant by inhibiting plant pathogenic fungi and promoting plant-beneficial microorganisms (Qin and Tian, 2005; Bekker et al., 2006; Bi et al., 2006). For example, potassium silicate inhibited the mycelial growth of *F. oxysporum* Schltdl.em. W. C. Snyder & H. N. Hanscn, a pathogenic fungi of tomato (Bekker et al., 2006); sodium metasilicate promoted the population of plant-beneficial microorganism *Cryptococcus laurentii* (Qin and Tian, 2005). Our results showed that sodium silicate increased the relative abundances of several microbial genera containing taxa with plant-growth-promoting and/or pathogen-inhibiting potentials, such as *Streptomyces* (Zhao et al., 2012), *Chaetomium* (Park et al., 2005), *Cryptococcus* (Qin and Tian, 2005), *Guehomyces* (Mestre et al., 2016), *Kernia* (Iwamoto et al., 1990) and *Pseudeurotium* spp. (Aseri et al., 2009) in bare soil, and *Rhodanobacter* (Thijs et al., 2014), *Guehomyces* (Mestre et al., 2016), and *Pseudeurotium* spp. (Aseri et al., 2009) in cucumber-cultivated soil. Moreover, sodium silicate decreased the relative abundances of microbial taxa containing potential plant pathogens, such *Stagonosporopsis* (Stewart et al., 2015) and *Nectria* spp. (Etebu and Osborn, 2010) in bare soil, and *Stagonosporopsis* (Stewart et al., 2015), *Ilyonectria* (Cabral et al., 2012) and *Myrothecium* spp. (Abbas et al., 2002) in cucumber-cultivated soil. These results also indicated that it was possible that sodium silicate exerted the positive feedback effects on cucumber growth and resistance through inhibiting plant pathogens and fostering beneficial microorganisms.

Sodium silicate at pH 9.85 inhibited FOC both in potato dextrose liquid medium when grown alone and in Gause’s synthetic liquid medium when grown with *Streptomyces* DHV3-2, but did not affect FOC in soil. These results only partially validated our second hypothesis, and suggested that sodium silicate may not decrease cucumber Fusarium wilt disease by directly inhibiting the growth of FOC in the soil environment. Mineral nutrients, such as zinc and copper, were shown to inhibit the *F. oxysporum* f.sp. *ciceri* to produce fusaric acid, a phytotoxin (Saikia et al., 2009), while some microorganisms, such as *Trichoderma harzianum*, could suppress fusaric acid produced by *Fusarium moniliforme*, a pathogen of maize (Elhasan et al., 2008). Therefore, there was a possibility that sodium silicate affected the pathogenicity of FOC directly or indirectly through changing soil microbial communities, which needs to be further elucidated.

*Streptomyces* spp. can protect plants through induction of host resistance (Palaniyandi et al., 2013). For example, Zhao et al. (2012) showed that culture filtrate from *S. bikiniensis* HD-087 induced systemic resistance in cucumber against Fusarium wilt. In the present study, enhanced activities of defense enzymes were observed in cucumber grown in the presence of soil biota from the treatment of cucumber-cultivated soil treated with sodium silicate. Moreover, sodium silicate at pH 9.85 had stimulatory effects on *Streptomyces* DHV3-2 in the soil environment. Illumina Miseq sequencing also showed that sodium silicate increased the relative abundance of *Streptomyces* spp. This further indicated that sodium silicate may reduce cucumber Fusarium wilt disease through stimulating plant-beneficial soil microorganisms which can induce systemic resistance in cucumber. Previous studies demonstrated that some mineral nutrients were able to improve the biocontrol efficiency of plant-beneficial microbes (Qin and Tian, 2005; Saikia et al., 2009). For example, zinc and copper stimulated *P. fluorescens* 4-92 to produce 2, 4-diacetylphloroglucinol, a secondary metabolite that can induce systemic resistance in plants (Saikia et al., 2009). Future studies should address if sodium silicate can enhance biocontrol activities of *Streptomyces* spp.

Sodium silicate at pH 9.85 inhibited FOC and *Streptomyces* DHV3-2 when they grown alone, and inhibited *Streptomyces* DHV3-2 in Gause’s synthetic liquid medium when these two species grown together. However, sodium silicate solution at pH 6.32 had no effects on FOC and *Streptomyces* DHV3-2. Therefore, our results validated the notion that the effect of pH played some role in the toxic effects of silicates on microorganisms (Bekker et al., 2006). We also found inconsistent effects of sodium silicate on FOC and *Streptomyces* DHV3-2 in synthetic medium and soil. For example, sodium silicate at pH 9.85 inhibited FOC in potato dextrose liquid medium but had no significant effect on FOC in soil; sodium silicate at pH 9.85 inhibited *Streptomyces* DHV3-2 in Gause’s synthetic liquid medium but promoted *Streptomyces* DHV3-2 in soil. This may due to the fact that carbon source and mineral nutrients, which differed between synthetic medium and soil, can affect the growth and physiological status of microorganisms (Jonsbu et al., 2002; Saikia et al., 2009).

## Conclusion

Overall, our results revealed that exogenous sodium silicate promoted cucumber seedling growth while decreased Fusarium wilt disease. Sodium silicate also changed soil bacterial and fungal community abundances, diversities, and compositions. However, soil microbial communities responded differently to sodium silicate in bare soil and cucumber-cultivated soil. Plant–soil feedback experiments showed that soil biota from cucumber-cultivated soil (but not bare soil) treated with sodium silicate enhanced cucumber seedling resistance to Fusarium wilt. These suggested that sodium silicate reduced Fusarium wilt disease through a cascade of changes in soil microbial communities, which may need the involvement of plants to exert these effects. Moreover, sodium silicate promoted the relative abundances of several microbial genera containing taxa with plant-growth-promoting and/or pathogen-inhibiting potentials, such as *Guehomyces* and *Pseudeurotium* spp. Further investigations are required to elucidate the role of these microbes in the enhanced cucumber resistance induced by sodium silicate.

## Author Contributions

FW and XZ designed the experiment. YS executed the experiments and wrote the manuscript. XF helped to polish the language. All authors reviewed the manuscript.

## Conflict of Interest Statement

The authors declare that the research was conducted in the absence of any commercial or financial relationships that could be construed as a potential conflict of interest.
