# Supplementary material for: Application of Sodium Silicate Enhances Cucumber Resistance to Fusarium Wilt and Alters Soil Microbial Communities
Source: Front Plant Sci. 2018 May 11;9:624. doi: 10.3389/fpls.2018.00624 (PMC5958222; doi:10.3389/fpls.2018.00624)

**Application of sodium silicate enhances cucumber resistance to Fusarium wilt and alters soil microbial communities**

Xingang Zhou^1,2^, Yanhui Shen^1^, Xuepeng Fu^1^, Fengzhi Wu^1,2*^

^1^Department of Horticulture, Northeast Agricultural University, Harbin, China

^2^ Key Laboratory of Biology and Genetic Improvement of Horticultural Crops (Northeast Region), Ministry of Agriculture, Harbin, China

Correspondence and requests for materials should be addressed to F.W. (fzwu2006@aliyun.com).

**Figure legends**

**FIGURE S1** Relative abundances of main soil bacterial (A) and fungal (B) phyla. For both bacterial and fungal communities, phyla with average relative abundances >1% were shown. BSi and B represent bare soil treated with and without sodium silicate, respectively. CSi and C represent cucumber-cultivated soil treated with and without sodium silicate, respectively.

**FIGURE S2** Heat maps showing the relative abundances of dominant classified bacterial (A) and fungal (B) genera. Classified bacterial genera with average relative abundances >0.5% and fungal genera with average relative abundances >0.3% were identified in each sample by colors deduced from the raw Z-scores. Hierarchical clustering of samples was performed using the average clustering method with the Euclidean distances. BSi and B represent bare soil treated with and without sodium silicate, respectively. CSi and C represent cucumber-cultivated soil treated with and without sodium silicate, respectively.

**FIGURE S1**


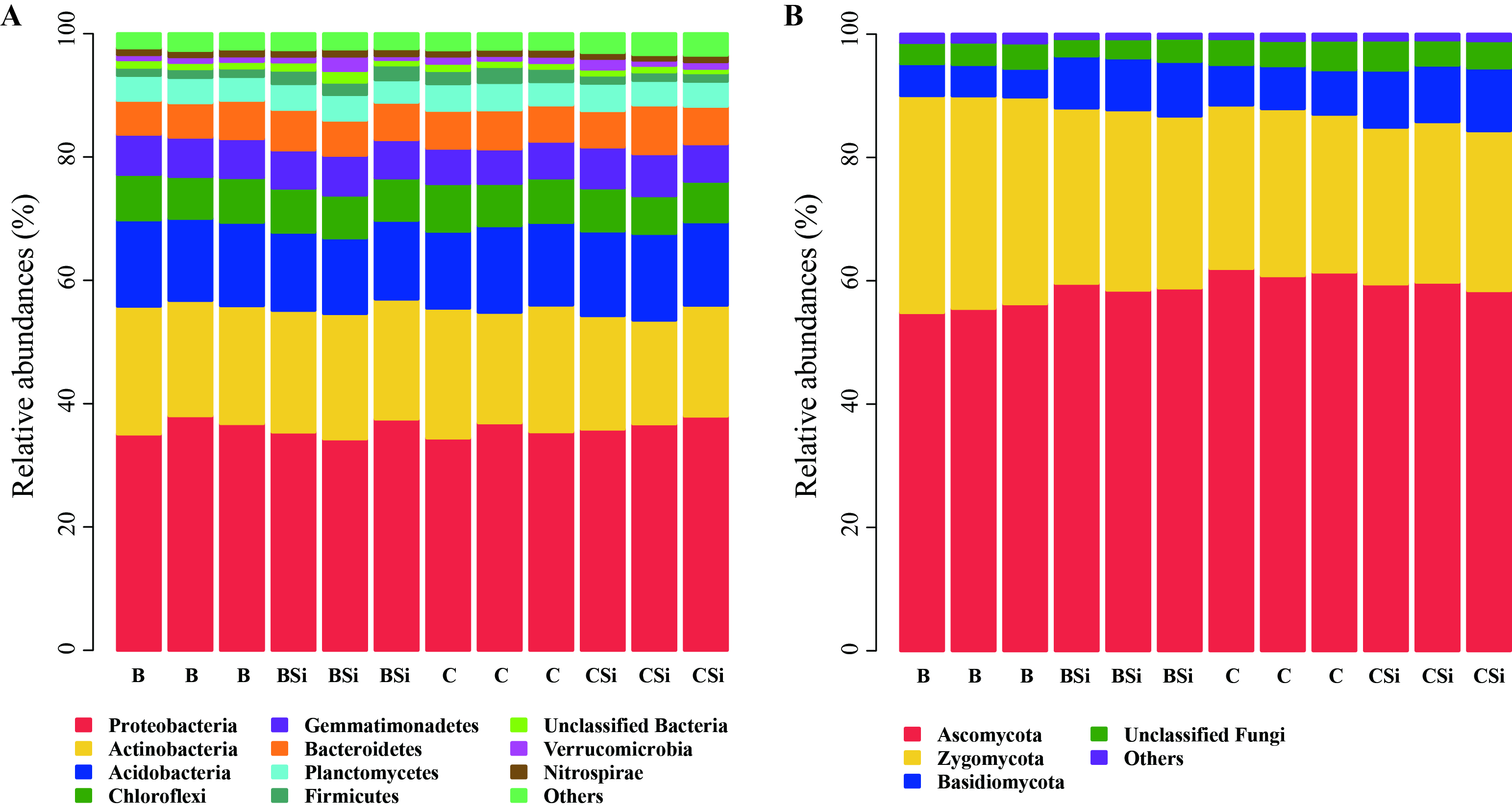


**FIGURE S2**


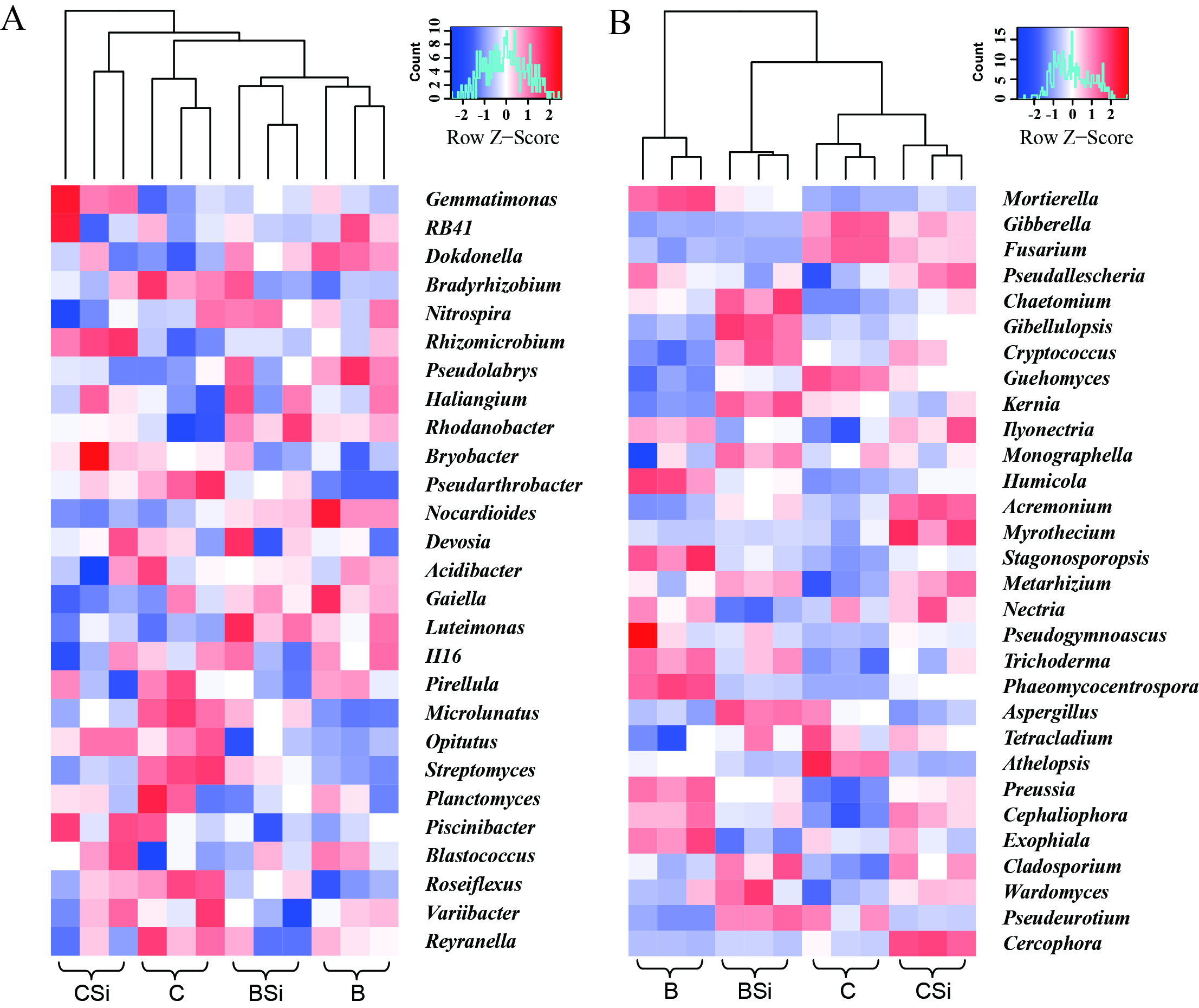

Supplement: Supplementary file 1 [file Data_Sheet_1.doc]
